# Ovarian Cysts in Polycystic Ovary Syndrome

**DOI:** 10.1001/jamainternmed.2026.1370

**Published:** 2026-05-11

**Authors:** Terhi T. Piltonen, Ewelina Kuusiniemi, Helena Teede

**Affiliations:** 1 Department of Obstetrics and Gynecology, Research Unit of Clinical Medicine, Medical Research Centre, University of Oulu, Oulu University Hospital, Oulu, Finland; 2 Monash Centre for Health Research and Implementation, Monash University, Melbourne, Australia

## Abstract

This cross-sectional study examines ovarian morphology regarding polycystic ovary syndrome.

Polycystic ovary syndrome (PCOS) is a common endocrine condition with multisystem manifestations, including metabolic, reproductive, dermatological, and psychological health implications^1,2^ (Figure). The syndrome affects 1 of 8 women, about 170 million reproductive-aged women globally and 10 million in the US.

**Figure.**
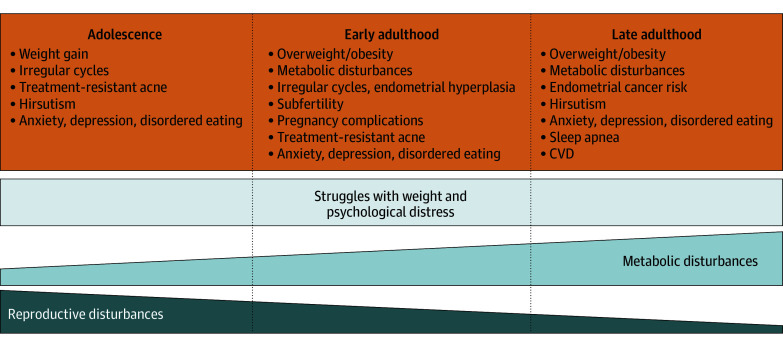
Visual Representation of Clinical Presentations of Polycystic Ovary Syndrome (PCOS) Common clinical presentations of PCOS at different stages of life show multiorgan involvement, supporting a holistic approach. CVD indicates cardiovascular disease.

Women with PCOS and their clinicians often mistakenly believe that PCOS is associated with pathological ovarian cysts. In a global survey with 7000 respondents, 85% of patients and 62% of clinicians associated PCOS with ovarian cysts.^3^ However, PCOS was named based on women with the syndrome having an excess of small antral follicles rather than benign or pathological noncancerous ovarian cysts, which are typically larger, epithelially lined, and may cause pain, torsion, rupture, or hemorrhage requiring surgical intervention. To address this common misperception and support the current effort to change the syndrome’s name, it would be helpful to understand how often women with PCOS have benign or pathological cysts. Therefore, we examined ovarian morphology in a prospective, population-based study.

## Methods

Data were derived from the prospective Women’s Health Study,^4^ which enrolled and examined 1918 participants aged 33 to 37 years between May 2020 and October 2022 at 2 university clinics. Participants provided written informed consent, and the ethical comittee of the County of North Ostrobothnia approved the study.

PCOS diagnosis was defined according to the Rotterdam criteria and current international guidelines.^2^ All participants underwent transvaginal ultrasonography to detect small antral follicles (2-9 mm), ovarian volume, ovarian (dominant) follicles (10-24 mm), and corpus luteum (CL) associated with the ovulatory cycle. PCOS-related multifollicular ovary was defined as 20 or more antral follicles in at least 1 ovary. Additionally, benign or pathological noncancerous ovarian cysts, including endometriomas and simple, paraovarian, hemorrhagic, or dermoid cysts were identified. Diagnostic and ovarian morphology assessments are described in the eMethods in Supplement 1.

Categorical variables were compared using the χ^2^ test or Fisher exact test, as appropriate. Odds ratios and 95% CIs were estimated using logistic regression with PCOS as a single covariate. Statistical analyses were performed using SPSS, version 29 (IBM). Statistical significance was set at 2-sided *P* ≤ .05.

## Results

Of 1904 Finnish (2.2% Sami individuals, 97.6% White European, and 0.2% other or not known) women aged 33 to 37 years (mean [SD] age, 35.3 [0.6] years), 1591 (83.6%) were categorized as not having PCOS and 313 (16.4%) received a diagnosis of PCOS. After excluding women who were using hormonal contraceptives, 1235 remained, of whom there were 1012 (81.9%) without PCOS and 223 (18.1%) with PCOS. Women with PCOS had 11.8-fold higher odds of having a multifollicular ovary (95% CI, 8.4-16.6) and 12.2-fold higher odds (95% CI, 8.3-17.9) of an ovarian volume of more than 10 mL compared with women without PCOS. There were no differences between the groups in the prevalence of ovarian (dominant) follicles, endometriomas, or simple, paraovarian, hemorrhagic, or dermoid cysts (Table). Even though almost one-third of women with PCOS were found to have CL, prevalence was lower than in women without PCOS.

**Table.** PCOS Diagnosis–Related and Other Benign Ovarian Findings in Women With and Without PCOS

Finding	No. (%)		OR (95% CI)
	Without PCOS (n = 1012)	PCOS (n = 223)	
PCOS-related			
≥20 Follicles (2-9 mm)^a^	117 (12.2)	133 (62.1)	11.83 (8.44-16.58)
Ovarian volume ≥10 mL^a^	53 (5.4)	91 (40.8)	12.18 (8.29-17.89)
Ovulation-related			
Dominant follicle (10-24 mm)	338 (33.6)	63 (28.3)	0.78 (0.57-1.07)
Corpus luteum^b^	361 (37.2)	60 (27.6)	0.65 (0.47-0.90)
Benign cysts			
Simple cyst (≥25 mm)	29 (3.0)	9 (4.1)	1.36 (0.64-2.91)
Paraovarian cyst	43 (4.4)	10 (4.5)	1.03 (0.51-2.08)
Hemorrhagic cyst	37 (3.7)	5 (2.2)	0.59 (0.23-1.51)
Pathological cysts			
Endometrioma	25 (2.5)	4 (1.6)	0.69 (0.24-2.00)
Dermoid cyst	8 (0.8)	4 (1.8)	2.29 (0.68-7.67)

Abbreviations: OR, odds ratio; PCOS, polycystic ovary syndrome.

^a^ At least in 1 ovary.

^b^ Included corpus luteum and luteinized unruptured follicles.

## Discussion

This cross-sectional study demonstrated that PCOS is not associated with an increased prevalence of benign or pathological noncancerous ovarian cysts. The relatively high number of CLs in women with PCOS suggests spontaneous ovulations; however, the fact that CL was less prevalent in women with than without PCOS suggests that ovulatory dysfunction among women with PCOS persists into the mid 30s.^5^ The findings also indicate that women with PCOS do not require ultrasonography more often than women without PCOS to detect benign or pathological noncancerous ovarian cysts. Given this, the diagnosis of PCOS can be based on physical findings and symptoms, and first-line management of symptoms and comorbidities can be managed by primary care clinicians using a holistic approach.^2^

This study compared ovarian morphology in women with PCOS with those without a diagnosis was strengthened by the use of a relatively large, unselected population, standardized methods, and modern ultrasonography. Limitations included a racially homogenous population with ultrasonography performed on random cycle days and by 5 different clinicians.

The findings of this cross-sectional study provide evidence that the concern of pathological ovarian cysts in PCOS is unfounded. This study provides more accurate, evidence-based information for patient care and supports a global process to change the condition’s name.

## Notes

Supplement 1. eMethods

Supplement 2. Nonauthor collaborators

Supplement 3. Data sharing statement
