# Supplementary material for: Ovarian Cysts in Polycystic Ovary Syndrome
Source: JAMA Intern Med. 2026 May 11;186(8):1041–3. doi: 10.1001/jamainternmed.2026.1370 (PMC13162137; doi:10.1001/jamainternmed.2026.1370)
Supplement: Supplement 1. — eMethods [file jamainternmed-e261370-s001.pdf]

Supplemental Online Content

Piltonen T, Kuusiniemi E, Teede H; Women's Health Study Research Group. Ovarian cysts in polycystic ovary syndrome. *JAMA Intern Med*. Published online May 11, 2026. doi:10.1001/jamainternmed.2026.1370

eMethods.

eReferences.

This supplemental material has been provided by the authors to give readers additional information about their work.

© 2026 Piltonen T et al. *JAMA Internal Medicine*.

eMethods.

The data were derived from the prospective Women's Health Study (WENDY), a population-based cohort study conducted in Finland between May 2020 and October 2022.[1] As previously published, with its birth cohort and national registry linkages, WENDY produced a large prospective population-level dataset on women's health. The WENDY dataset includes 1,918 women born in 1986–1987 in northern Finland who were 33–37 years old at the time of the study visit. The study aim was to capture gynecological, metabolic, and overall health outcomes in reproductive-aged women.

All participants underwent transvaginal ultrasound examinations (System HS60, Samsung Healthcare, Seoul, South Korea) using an 11 MHz transducer, performed by five gynecologists at the Oulu and Helsinki University Hospitals on a random day of the menstrual cycle. Written informed consent was obtained from all participants prior to their participation. Ethical approval was granted by the ethics committees of the Northern Ostrobothnia (decision numbers NFBC-86: 108/2017; WENDY: 49/2019) and the Helsinki and Uusimaa Hospital Districts (decision number 483/2020).

PCOS was defined in accordance with current guidelines,[2] which require at least two of the following: self-reported oligo-amenorrhea, PCOS-related ovarian morphology, and/or signs of hyperandrogenism. Oligo-amenorrhea was defined as a menstrual cycle length of >35 or <21 days, or fewer than eight cycles annually. Real-time analysis of PCOS-related ovarian morphology was prospectively performed during the WENDY study visit. A small antral follicle (2-9mm) count ≥ 20 per ovary (multifolliclar ovary, MFO) or an ovarian volume ≥ 10 mL was considered diagnostic. Hyperandrogenism was assessed using the modified Ferriman-Gallwey score, with a score of ≥4 considered diagnostic of hirsutism, as recommended by international guidelines.[2] Alternatively, serum testosterone of >1.8 nmol/L or free-androgen index >4.5 was considered a diagnostic sign of biochemical hyperandrogenism. Serum sex hormone–binding globulin (SHBG) was measured by immunochemiluminometric assay, and serum testosterone with liquid chromatography-mass spectrometry.[1] Other potential causes of menstrual irregularity, hyperandrogenism, or related symptoms were systematically excluded using laboratory testing and clinical evaluation (i.e., testing for hypothyroidism and hyperprolactinemia and excluding women lactating during the previous 3 months and those who were pregnant during the past 6 months). Participants using any form of hormonal contraception were excluded from the analysis. This included combined estrogen–progestin formulations, progestin-only therapy, and progestin-releasing intrauterine devices. After all these exclusions, 18.1% of women were considered as having PCOS according to the Rotterdam criteria; of them 68.6% (n=153) had the diagnosis based on ultrasonography.

Ovarian structures were documented based on morphological assessments on ultrasound, including Doppler imaging. Dominant follicles, defined as 10–24 mm in diameter and a part of the normal menstrual cycle, were interpreted with

© 2026 Piltonen T et al. *JAMA Internal Medicine*.

reference to cycle phase and endometrial status. The corpus luteum was identified based on morphology and vascular status by Doppler imaging, cycle phase, and endometrial status. Luteinized unruptured follicles were not separated from the corpus luteum, as both are considered a part of the normal menstrual cycle.

Benign cysts were reported as simple cysts, which were defined as ≥25 mm in diameter, paraovarian cysts were of various sizes adjacent to the ovary, and hemorrhagic cysts were identified by the presence of internal hemorrhagic components. Pathological, non-cancerous ovarian findings, endometriomas and dermoid cysts, were classified based on classical appearance and confirmed by a second opinion.

Classifications were made in real time, and in cases of uncertainty, retrospective evaluation was supplemented by laboratory testing and a second opinion. Repeated analysis and still- or 3D-image re-review was performed only when clinically relevant (for endometriomas or dermoid cysts). Images with poor quality or in which ovaries were not visualized were excluded.

Statistical analyses were performed using IBM SPSS Statistics software (IBM Corp., Armonk, NY, USA, version 29.0). Categorical variables were compared using the χ^2 test or Fisher's exact test, as appropriate. Associations were further quantified using logistic regression to calculate odds ratios (ORs) with 95% confidence intervals (CIs). Statistical significance was set at $P \leq 0.05$.

eReferences

1. Piltonen TT, Ohtamaa M, Arffman RK, et al. Women's Health Study (WENDY)—a protocol of a population-based study assessing gynecological and metabolic health in women in their mid-30s. *Am J Epidemiol.* 2025 Mar 4;194(3):598-607.
2. Teede HJ, Tay CT, Laven JJE, et al. Recommendations from the 2023 International Evidence-based Guideline for the Assessment and Management of Polycystic Ovary Syndrome. *J Clin Endocrinol Metab.* 2023 Sep 18;108(10):24472469.

© 2026 Piltonen T et al. *JAMA Internal Medicine*.
