# Supplementary material for: Ovarian Cysts in Polycystic Ovary Syndrome
Source: JAMA Intern Med. 2026 May 11;186(8):1041–3. doi: 10.1001/jamainternmed.2026.1370 (PMC13162137; doi:10.1001/jamainternmed.2026.1370)
Supplement: Supplement 2. — Nonauthor collaborators [file jamainternmed-e261370-s002.pdf]

Supplemental Online Content: Nonauthor Collaborators
*First name, last name, and suffix (if applicable) are required and will appear in PubMed.

*Group Name(s): The WENDY Research Group							
***First Name and Middle Initial(s)**	***Last Name**	***Suffix (eg, Jr, III)**	Academic Degrees	Institution	Location (city, state/province, country)	Role or Contribution, eg, chair, principal investigator	Group (if more than 1 Group listed in the byline) and/or Subgroup (eg, Steering Committee)
Riikka	Arffman		PhD	Department of Obstetrics and Gynecology, Research Unit of Clinical Medicine, Medical Research Centre, University of Oulu, Oulu University Hospital	Oulu, Finland	funding acquisition, project administration, data curation, review & editing	
Elisa	Hurskainen		MSc	Department of Obstetrics and Gynecology, Research Unit of Clinical Medicine, Medical Research Centre, University of Oulu, Oulu University Hospital	Oulu, Finland	data curation, review & editing	
Jari	Jokelainen		MSc	Northern Finland Birth Cohorts, Arctic Biobank, Infrastructure for Population Studies, Faculty of Medicine, University of Oulu	Oulu, Finland	data curation, project administration, review & editing	
Marika	Kangasniemi		MD, PhD	Department of Obstetrics and Gynecology, Research Unit of Clinical Medicine, Medical Research Centre, University of Oulu, Oulu University Hospital	Oulu, Finland	investigation, review & editing	
Jenni	Kinnunen		MD, PhD	Department of Obstetrics and Gynecology, Research Unit of Clinical Medicine, Medical Research Centre, University of Oulu, Oulu University Hospital	Oulu, Finland	investigation, review & editing	
Aino	Kurki		BM	Department of Obstetrics and Gynecology, Research Unit of Clinical Medicine, Medical Research Centre, University of Oulu, Oulu University Hospital	Oulu, Finland	data curation, review & editing	

Supplemental Online Content: Nonauthor Collaborators
*First name, last name, and suffix (if applicable) are required and will appear in PubMed.

***First Name and Middle Initial(s)**	***Last Name**	***Suffix (eg, Jr, III)**	Academic Degrees	Institution	Location (city, state/province, country)	Role or Contribution, eg, chair, principal investigator	Group (if more than 1 Group listed in the byline) and/or Subgroup (eg, Steering Committee)
Kaisu	Luiro		MD, PhD	Department of Obstetrics and Gynecology, Reproductive Medicine Unit, Helsinki University Hospital, University of Helsinki,	Helsinki, Finland	investigation, review & editing	
Maria	Rajecki		MD, PhD	Department of Obstetrics and Gynecology, Reproductive Medicine Unit, Helsinki University Hospital, University of Helsinki,	Helsinki, Finland	investigation, review & editing	
Susanna	Savukoski		MD, PhD	Department of Obstetrics and Gynecology, Research Unit of Clinical Medicine, Medical Research Centre, University of Oulu, Oulu University Hospital	Oulu, Finland	investigation, review & editing	
