# Supplementary material for: Ovarian Cysts in Polycystic Ovary Syndrome
Source: JAMA Intern Med. 2026 May 11;186(8):1041–3. doi: 10.1001/jamainternmed.2026.1370 (PMC13162137; doi:10.1001/jamainternmed.2026.1370)
Supplement: Supplement 3. — Data sharing statement [file jamainternmed-e261370-s003.pdf]

Data Sharing Statement

Data

Data available: Yes
Data types: Deidentified participant data, Data dictionary
How to access data: https://www.oulu.fi/en/university/faculties-and-units/faculty-medicine/northern-finland-birth-cohorts-and-arctic-biobank/womens-health-study
When available: With publication

Supporting Documents

Document types: Other (please specify)
Additional Information: DTA
How to access documents: https://www.oulu.fi/en/university/faculties-and-units/faculty-medicine/northern-finland-birth-cohorts-and-arctic-biobank/womens-health-study
When available: With publication

Additional Information

Who can access the data: Researchers whose proposed use of the data has been approved and who follows the GDPR
Types of analyses: Academic purpose
Mechanisms of data availability: A signed data access agreement
Any additional restrictions: requested data is already under analysis
